# Practical Notes on Diagnosis and Treatment

**Published:** 1910-01-15

**Authors:** 


### Practical Notes on Diagnosis and Treatment.

**Tabes Dorsalis.**

Remember the ætiological association of aneurysm, angina, and tabes.—*Dr. F. W. Mott.*

**Oil of Cajuput.**

Two or three drops of oil of cajuput on a lump of sugar forms a very efficient carminative and antifermentative.—*Dr. Murell.*

**Adherent Pericardium.**

If a cardiac case presenting the features of mitral incompetence fails to improve with rest in bed and digitalis, think of adherent pericardium.—*Dr. J. Mitchell Bruce.*

**To Abort a Cold.**

The inhalation through the nostrils of formaldehyde vapour is very efficacious. This may be done conveniently by sniffing at the neck of a small flask containing formalin solution.

**Sore-throat.**

One of the earliest symptoms of the onset of acute nephritis may be a sore throat. Inspection generally reveals only a moderate congestion, with swelling of the fauces and uvula.

**Thread Worms.**

The internal administration of sulphur has been productive of excellent results, and should be given a trial before resorting to the inconvenient and often inefficiently given injections.

**Bright's Disease.**

Bear in mind the importance of maintaining the excretion of urine, although some attention may be paid to the excretory function of the skin. Remember an ounce of urine is worth a great deal of sweat.—*Dr. T. Henry Green.*

**Scarlet Fever.**

Desquamation confined to the palms and soles at the end of six weeks may be disregarded in considering the safety of discharging a patient convalescent from scarlet fever if he is free from any catarrh of ears or nose.—*Dr. B. Ker.*

**Sciatica.**

Careful examination of the corresponding hip joint should be made in all cases of sciatica, and a radiogram taken if possible. Most cases of sciatica seem to be associated with some arthritic change in the joint.—*Dr. William Bruce.*

**The Pupils and Arterial Tension.**

Contracted pupils are commonly found in conditions where the blood-pressure is raised, as in granular kidney. This may be explained by the fact that in the iris the arteries are spirally arranged, and hence when the arterial tension is raised these tend to straighten out, and so the iris contracts. Conversely, the pupils may be dilated in those conditions, accompanied by a low-tension pulse, as in chlorosis, except in the earlier stages, where, on account of the hydræmia, the tension may be slightly raised.

**Ulcers of the Leg.**

The encouraging results obtained by the administration of iodide of calcium in cases of chronic ulceration of the leg seem to be confirmed by the reports of later observers. The dose ranges from 2 to 5 grains, three times a day in a mixture. The drug is very deliquescent.

**Ankle-jerks.**

The loss of the ankle jerk (*tendo achillis reflex*) often precedes loss of the knee-jerk; this with difficulty or some derangement of the act of micturition should arouse suspicion of tabes dorsalis, even if the Argyll-Robertson pupil should not then be present.—*Dr. F. W. Mott.*

**Hæmatemesis.**

This is far from being always due to bleeding from a gastric ulcer. It is often met with in cases of anæmic gastritis. Remember also that the gastric ulcers which bleed are not those most likely to perforate; the previous histories of cases of perforated gastric ulcer are not infrequently devoid of definite symptoms.—*Dr. Hale White.*

**Auscultation in Heart Cases.**

It is sound advice not only to students, but also to practitioners, to say: Keep your stethoscope in your pocket as long as possible; exhaust all other means of gaining information, especially in heart cases, before listening through a stethoscope. Many cases can be quite well diagnosed without auscultation at all, *e.g.* aortic incompetence.—*Dr. T. Henry Green.*

**Sulphate of Soda.**

This drug is not nearly so often used by the general practitioner as the rival magnesium salt, though its merits and advantages warrant a greater vogue for it. It is more pleasant to take, and not so violent nor so griping in its action as Epsom Salts. It has been highly spoken of in the treatment of those small boils which so frequently crop out on the neck at the level of the edge of the collar.

**Hæmoptysis and Amyl Nitrite.**

The treatment of hæmoptysis by ergot or by adrenalin is unsound and even dangerous. Far better would it be to do nothing except order absolute rest with or without morphia, according to the temperament of the patient. The inhalation of amyl nitrite, on the other hand, is sound therapeutics, as shown by direct laboratory experiment and by clinical experience.—*Dr. Francis Hare.*

**Cirrhosis of the Liver.**

Do not forget that cirrhosis of the liver is frequently associated with tuberculosis, especially of the lungs and peritoneum.—*Dr. Newton Pitt.*

Cases of so-called cirrhosis which have been subjected to several tappings for ascites are more properly described as cases of perihepatitis. True cirrhosis cases are not benefited by removal of the fluid, and usually die in a few months.—*Dr. Hale White.*

